# Pre- and post-treatment evaluation of routine blood analysis in patients with attention deficit hyperactivity disorder and comparison with the healthy control group

**DOI:** 10.1038/s41598-023-43553-5

**Published:** 2023-09-27

**Authors:** Erdoğan Öz, Mehmet Emin Parlak, Yaşar Kapıcı, Umut Balatacı, Osman Küçükkelepçe, Fatma Kurt

**Affiliations:** 1 Adıyaman Provincial Health Directorate, Adiyaman, Turkey; 2 Department of Pediatrics, Kepez State Hospital, Antalya, Turkey; 3 Department of Psychiatry, Adana 5 Ocak State Hospital, Adana, Turkey; 4 Department of Child and Adolescent Psychiatry, Kahta State Hospital, Adiyaman, Turkey; 5 Public Health Division, Adıyaman Provincial Health Directorate, Adiyaman, Turkey; 6 Department of Child and Adolescent Psychiatry, Adıyaman Training and Research Hospital, Adiyaman, Turkey

**Keywords:** Neuroscience, Psychology, Biomarkers

## Abstract

This study aimed to examine potential disparities in hematologic inflammation parameters between children diagnosed with attention deficit hyperactivity disorder (ADHD) and their healthy counterparts and to determine whether atomoxetine treatment induced any alterations in inflammation indicators. This case–control study involved 43 children aged 6–13 years, 22 diagnosed with ADHD for the first time, and 21 healthy children. In all children, complete blood count and albumin, C-reactive protein (CRP), thyroid stimulating hormone (TSH), free thyroxine (free T4), folate, vitamin B12, aspartate aminotransferase (AST), alanine transaminase (ALT), creatinine and urea values were performed. Children with ADHD were started on atomoxetine treatment, and one month later, the blood test was repeated for those who commenced treatment. Neutrophil (p = 0.005), platelet (PLT) (p = 0.002), neutrophil/lymphocyte ratio (NLR) (p = 0.001), platelet/lymphocyte ratio (PLR) (p < 0.001), systemic immune /inflammation index (SII) (p < 0.001) and pan-immune-inflammation value (PIV) (p = 0.025) parameters were found to be significantly higher than the control group, while the lymphocyte value (p = 0.001) was found to be significantly lower. In those in the ADHD group, lymphocyte (p = 0.041) and albumin (p = 0.027) values increased significantly after treatment. The results of this study show the increase in inflammation in drug-naive ADHD patients and the partial improvement after treatment. However, there is a need to evaluate inflammation in larger samples after longer-term treatments and follow-ups.

## Introduction

ADHD is a neurodevelopmental disorder primarily observed in childhood, characterized by symptoms such as inattention, excessive activity, and impulsivity that are inconsistent with typical developmental patterns^1^. The prevalence of ADHD tends to decrease with age, but it persists into adulthood in approximately two-thirds of cases^2,3^. Although studies conducted in different parts of the world have reported significantly different results, the worldwide-pooled prevalence of ADHD is estimated to be 5.29%^4^.

Inflammation has been found to be associated with neuropsychiatric disorders not only in adults but also in children and adolescents. Studies have shown that markers of inflammation increase in various neuropsychiatric conditions in these age groups^5,6^. Moreover, investigations exploring the connection between ADHD and peripheral inflammation have highlighted the potential role of neuroinflammation in the pathophysiology of ADHD. Animal studies have also provided evidence suggesting a relationship between exposure to inflammation and the development of ADHD^6^.

Leukocytes, neutrophils, and platelets play an essential role in inflammation. CRP and albumin also respond to inflammation as acute-phase reactants^7^. NLR from systemic inflammation indicators and PLR from chronic inflammation indicators; increases in psychiatric disorders such as ADHD, schizophrenia, bipolar and obsessive–compulsive disorder^1,8–11^. Similarly, it has been shown that the monocyte/ lymphocyte ratio (MLR) is high in psychiatric disorders^8–10^.

In addition to NLR, PLR, and MLR, it has been reported that CRP/Albumin ratio (CAR), Red Cell Distribution Width (RDW), and Mean Platelet volume (MPV) are high in schizophrenia patients^7^. Similar to CRP, SII ((platelet count x neutrophil count)/lymphocyte count) is a superior indicator of inflammation than NLR, PLR, and MLR. Nitric oxide, arginase, and reactive oxygen species released in the presence of high SII may play a role in the development of ADHD by inhibiting activated T cells^12^. It has been found that PIV ((platelet count x neutrophil count x monocyte count)/lymphocyte count) is high in obsessive–compulsive disorder^13^.

Indeed, some studies have reported elevated levels of neutrophils alone and platelets (PLT) alone in individuals with ADHD compared to control groups^14,15^. Conversely, the lymphocyte level is lower in individuals with ADHD^9^. Additionally, there is a suggested relationship between ADHD and decreased levels of albumin^16^. These findings further support the potential involvement of these specific hematologic parameters, such as neutrophils, platelets, lymphocytes, and albumin, in the pathophysiology of ADHD.

The treatment options for ADHD include pharmacological interventions, behavioral therapy, or a combination. One of the pharmacological treatment options is atomoxetine, a selective norepinephrine (NE) reuptake inhibitor. Atomoxetine is an approved medication for ADHD in children, adolescents, and adults. Although the precise mechanism of action of atomoxetine remains unknown, it is known to inhibit the reuptake of dopamine in specific brain regions while preventing NE reuptake. Notably, atomoxetine significantly improves ADHD symptoms within a few weeks of treatment. Moreover, atomoxetine has shown effectiveness in addressing symptoms associated with comorbidities often observed in individuals with ADHD^17^.

Based on the provided references, there is limited information about direct or indirect evidence explicitly addressing the effect of atomoxetine on inflammatory markers in individuals with ADHD. Further research explicitly investigating this aspect is needed to determine the impact of atomoxetine on inflammatory markers in the context of ADHD treatment.

The primary objective of this study was to examine potential differences in hematologic inflammation parameters between children diagnosed with ADHD and healthy children without any complaints. Additionally, the study aimed to determine if these parameters could serve as diagnostic markers for ADHD. Furthermore, the study investigated whether atomoxetine treatment impacted the inflammation indicators.

## Methods

### Ethics approval

Ethical approval for the study was obtained from the Fırat University Non-Interventional Research Ethics Committee, dated December 1, 2022, with the reference number 2022/14-05. Since all participants in the study were under the age of 18, written consent was obtained from their families, indicating their agreement to participate in the study. Furthermore, all procedures adhered to the principles outlined in the Declaration of Helsinki and complied with relevant local laws and regulations.

### Study setting

This case–control study was conducted at the Kahta District State Hospital in the Southeastern Anatolia Region of Turkey, from January 1, 2023, to May 1, 2023. The study included two groups, the case and the control groups, with similar sociodemographic characteristics. The case group consisted of children aged 6–13 years who presented at the Child and Adolescent Psychiatry Outpatient Clinic and received a first-time diagnosis of ADHD based on the diagnostic criteria outlined in the DSM-V. In contrast, the control group comprised children without complaints, acute or chronic illness, or concurrent medication use who attended the Healthy Child Outpatient Clinic for routine screening blood tests.

G*Power version 3.1.9.2 program was used to calculate the sample size in the study. For sample calculation, Önder et al. study was referenced^18^. When calculating the sample size, when 0.5 effect size, 5% margin of error, and 80% power were accepted, it was planned to include 38 children, 19 for the case group and 19 for the control group.

All blood samples were collected via venous blood sampling. Following a 12-h fasting period, a comprehensive set of blood tests, including CBC, albumin, CRP, TSH, free T4, folate, vitamin B12, AST, ALT, creatinine, and urea values, were conducted in all participating children. For children diagnosed with ADHD, atomoxetine treatment was initiated as a single daily dose in the morning. The initial atomoxetine dose was set at 0.5 mg per kg per day for the first week, followed by a maintenance dose of 1.2 mg per day after one week^17^. The maximum daily dose of atomoxetine administered to any treated child did not exceed 80 mg.

4 of the 48 ADHD patients examined at the Child and Adolescent Psychiatry Outpatient Clinic were not included in the study because their parents did not have consent. Among the remaining patients, those with additional psychiatric diagnoses of autism spectrum disorder (3), mental retardation (1), obsessive–compulsive disorder (2), oppositional defiant disorder (2), and depression (1) were excluded from the study.

**Figure 1:**
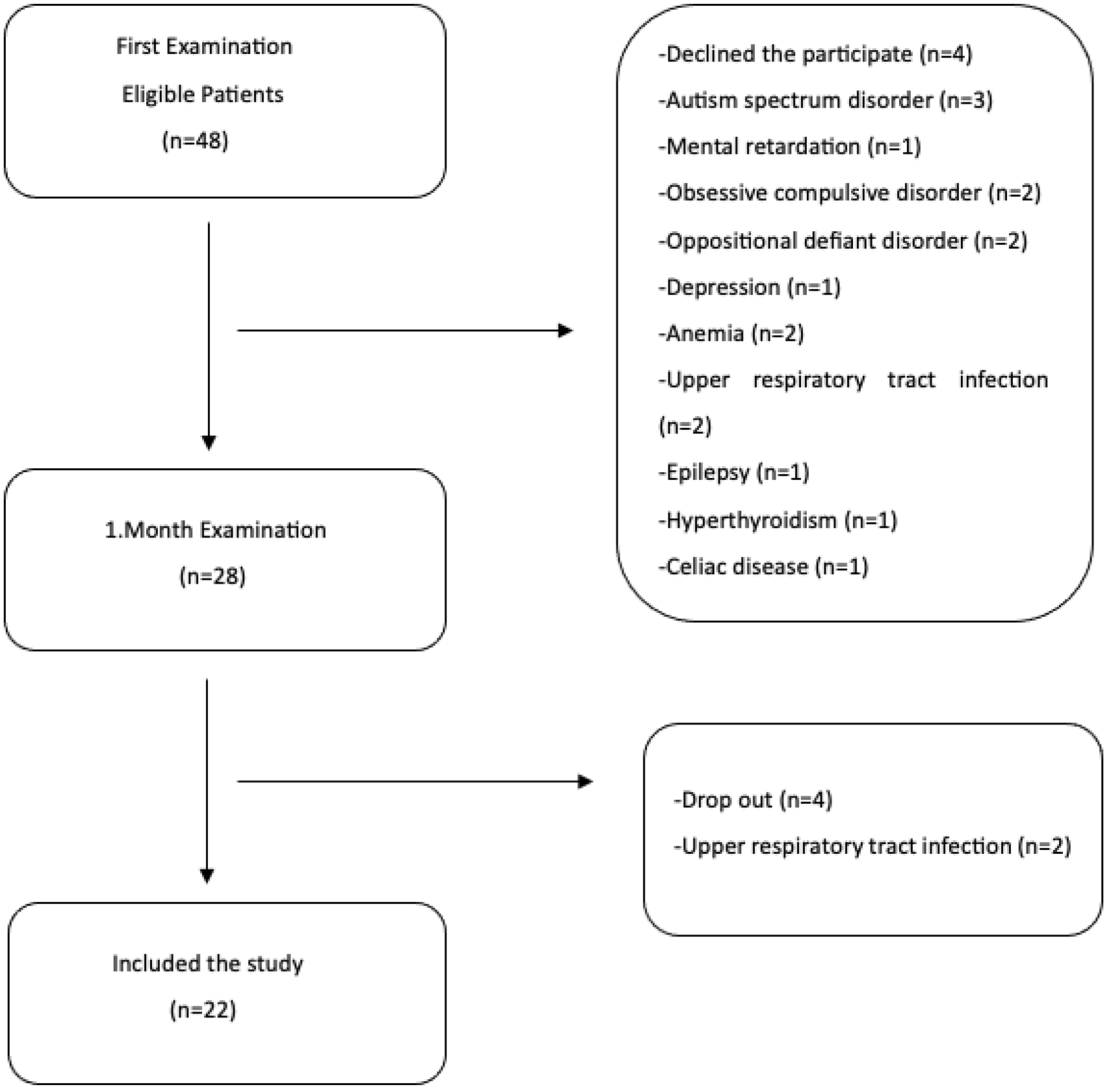
Flow-chart illustration of the study’s sample.

In order to evaluate the comorbidities of the ADHD patients and the control group, they were examined by a specialist pediatrician at the pediatric outpatient clinic at their first admission and 1-month follow-up. Among ADHD patients, those with anemia (2), upper respiratory tract infection (2), epilepsy (1), hyperthyroidism (1), and celiac disease (1) were not included in the study. After one month of atomoxetine treatment, the children underwent a follow-up visit for evaluation, and blood tests were repeated. 4 ADHD patients who did not come to follow-up at the 1-month follow-up and 2 ADHD patients with upper respiratory tract infections were excluded from the study. Flow-chart illustration of the study’s sample was shown in Fig. 1.

### Statistics

Statistical analyses were conducted using the SPSS software package (SPSS Inc., Chicago, IL) version 22. Descriptive statistics were presented as n and % for categorical data, and mean ± standard deviation (mean ± SD) or median with interquartile range (25th-75th percentile values) for continuous data. Categorical variables were compared between groups using the Pearson chi-square test, and the Shapiro–Wilk test was used to assess the normality of continuous variables. Paired groups were compared using the Mann–Whitney U test, and values before and after treatment were compared using the Wilcoxon signed-rank test. Receiver operating characteristic (ROC) curves were generated to evaluate the diagnostic value of various parameters in diagnosing ADHD. The statistical significance level for all analyses was set at p < 0.05.

## Results

A total of 43 children participated in the study, with 22 children in the ADHD group and 21 children in the healthy control group. In the ADHD group, 36.4% were girls, and 63.6% were boys. In the control group, 47.6% were girls, and 52.4% were boys. There was no significant difference in gender distribution between the two groups (p = 0.712). The median age of the children in the ADHD group was 8.3 years (range: 7.0–9.3), with a mean age of 8.6 ± 2.2 years. In the control group, the median age was 5.0 years (3.4–13.6), with a mean age of 7.8 ± 5.1 years. There was no significant difference in age between the two groups (p = 0.307) (Table 1).

**Table 1:** Comparison of demographic characteristics by groups.

		ADHD (n = 22)		Control (n = 21)		P
		Number	%	Number	%	
Gender	Female	8	36.4	10	47.6	0.712*
	Male	14	63.6	11	52.4	
Age, median (IQR)		8.3 (7.0–9.3)		5.0 (3.4–13.6)		0.307**

*Chi-square analysis.

**Mann Whitney U test was applied.

The ADHD group showed significantly higher values of neutrophils (p = 0.005), PLT (p = 0.002), NLR (p = 0.001), PLR (p < 0.001), SII (p < 0.001), and PIV (p = 0.025) compared to the control group. Conversely, the lymphocyte value was significantly lower in the ADHD group (p = 0.001). No significant difference was detected between the two groups in the other blood parameters compared (Table 2).

**Table 2:** Comparison of the laboratory values of the groups.

	ADHD (n = 22)	Control (n = 21)	p*
	Median (IQR)	Median (IQR)	
Hgb (g/dL)	12.8 (12.5–13.6)	12.8 (11.9–13.5)	0.531
Neutrophil (%)	55.7 (48.2–72.9)	44.6 (40.1–53.6)	**0.005**
Lymphocyte (%)	26.5 (21.1–35.1)	43.5 (34.3–50.6)	**0.001**
Monocyte (%)	5.8 (2.2–6.8)	5.9 (4.0–7.2)	1.000
Basophil (%)	1.3 (1.1–1.3)	1.2 (0.8–1.8)	0.823
Eosinophil (%)	3.8 (1.9–6.0)	4.4 (2.3–5.9)	0.553
WBC(× 10^3^/μL)	8.6 (6.0–11.1)	8.0 (7.5–9.5)	0.667
PLT(× 10^3^/μL)	330.0 (297.2–423.7)	267.0 (248.0–294.8)	**0.002**
Albumin (g/dL)	4.2 (4.0–4.3)	4.1 (4.0–4.5)	0.653
CRP (mg/dL)	1.0 (0.2–1.5)	0.2 (0.2–1.3)	0.640
TSH (uIU/mL)	2.0 (1.8–2.2)	2.2 (1.4–3.2)	0.417
fT4 (ng/dL)	0.9 (0.8–0.9)	0.9 (0.8–1.0)	0.308
Folate (ng/mL)	12.9 (12.3–13.8)	10.5 (8.0–14.0)	0.492
Vitamin B12 (pg/mL)	185.0 (148.0–209.0)	162.5 (131.0–234.0)	0.724
MPV (fL)	6.7 (5.7–7.6)	7.0 (5.8–7.6)	0.845
PDW (f/L)	18.3 (16.9–19.4)	18.1 (17.2–19.4)	0.938
PCT (%)	0.2 (0.1–0.2)	0.2 (0.2–0.2)	0.088
RDW (%)	10.1 (9.8–18.0)	10.3 (9.7–12.2)	0.755
AST (U/L)	29.0 (24.0–35.0)	25.5 (21.0–30.9)	0.183
ALT (U/L)	16.0 (15.0–18.0)	14.0 (10.0–16.7)	0.183
Creatinine (mg/dL)	0.5 (0.4–0.5)	0.5 (0.4–0.6)	0.123
Urea (mg/dL)	25.0 (19.0–29.0)	24.0 (21.0–26.0)	0.938
NLR	2.0 (1.4–4.2)	1.0 (.7–1.7)	**0.001**
PLR	11.6 (8.1–19.6)	6.2 (5.0–7.7)	** < 0.001**
MLR	0.2 (0.1–0.3)	0.1 (0.1–0.2)	0.155
CAR	0.2 (0.0–0.5)	0.2 (0.0–0.7)	0.930
SII	644.9 (382.7–1295.3)	301.0 (184.0–409.6)	** < 0.001**
PIV	4392.1 (1267.8–9794.6)	1478.7 (966.4–2524.2)	**0.025**

*Mann Whitney U test was applied.

Significant values are in bold.

After treatment, the ADHD group showed a significant increase in lymphocyte values (p = 0.041) and albumin values (p = 0.027). No significant difference was detected in the other blood parameters examined. (Table 3).

**Table 3:** Comparison of pre-treatment and post-treatment laboratory values in the ADHD group.

	Before treatment	After treatment	p*
	Median (IQR)	Median (IQR)	
Hgb (g/dL)	12.8 (12.5–13.6)	13.2 (12.6–14.8)	0.091
Neutrophil (%)	55.7 (48.2–72.9)	45.7 (38.8–59.0)	0.091
Lymphocyte (%)	26.5 (21.1–35.1)	39.2 (32.3–53.9)	**0.041**
Monocyte (%)	5.8 (2.2–6.8)	6.0 (4.0–9.2)	0.657
Basophil (%)	1.3 (1.1–1.3)	0.9 (0.5–1.6)	0.594
Eosinophil (%)	3.8 (1.9–6.0)	2.0 (1.6–4.3)	0.248
WBC(× 10^3^/μL)	8.6 (6.0–11.1)	9.5 (7.1–13.2)	0.182
PLT(× 10^3^/μL)	330.0 (297.2–423.7)	339.9 (274.0–382.1)	0.859
Albumin (g/dL)	4.2 (4.0–4.3)	4.3 (4.1–4.5)	**0.027**
CRP (mg/dL)	1.0 (0.2–1.5)	0.7 (0.3–1.4)	0.917
TSH (uIU/mL)	2.0 (1.8–2.2)	2.7 (1.8–4.1)	0.139
fT4 (ng/dL)	0.9 (0.8–0.9)	0.9 (0.8–1.0)	0.248
Folate (ng/mL)	12.9 (12.3–13.8)	13.7 (10.1–18.4)	0.918
Vitamin B12 (pg/mL)	185.0 (148.0–209.0)	274.0 (190.0–484.0)	0.116
MPV (fL)	6.7 (5.7–7.6)	6.7 (6.0–7.2)	0.477
PDW (f/L)	18.3 (16.9–19.4)	18.4 (17.6–19.1)	0.790
PCT (%)	0.2 (0.1–0.2)	0.2 (0.2–0.2)	0.075
RDW (%)	10.1 (9.8–18.0)	10.4 (9.8–12.2)	0.799
AST (U/L)	29.0 (24.0–35.0)	28.0 (24.0–35.0)	0.720
ALT (U/L)	16.0 (15.0–18.0)	17.0 (15.0–18.0)	0.384
Creatinine (mg/dL)	0.5 (0.4–0.5)	0.4 (0.4–0.5)	0.497
Urea (mg/dL)	25.0 (19.0–29.0)	22.5 (17.0–29.0)	0.859
NLR	2.0 (1.4–4.2)	1.1 (0.7–1.8)	0.131
PLR	11.6 (8.1–19.6)	8.3 (6.6–11.8)	0.131
MLR	0.2 (0.1–0.3)	0.2 (0.1–0.3)	0.594
CAR	0.2 (0.0–0.5)	0.2 (0.1–0.3)	0.686
SII	644.9 (382.7–1295.3)	352.6 (230.1–661.5)	0.091
PIV	4392.1 (1267.8–9794.6)	2105.5 (1246.2–5108.7)	0,248

*Wilcoxon analysis was applied.

Significant values are in bold.

ROC analysis was conducted to assess the predictive ability of various values for ADHD diagnosis, and cut-off values were determined. The results indicated that neutrophil, lymphocyte, PLT, NLR, PLR, SII, and PIV parameters had a significant diagnostic value for ADHD (Table 4).

**Table 4:** The specificities and sensitivities of the measured parameters in determining the diagnosis of ADHD.

	Area	p	95% confidence interval		Sensitivity	Specificity	PPD	NPD
			Lower limit	Upper limit				
Neutrophil > 46.39	0.801	** < 0.001**	0.622	0.920	90.9	61.9	55.6	92.9
Lymphocyte ≤ 39.59	0.835	** < 0.001**	0.662	0.942	90.9	66.7	58.8	93.3
PLT > 294.8	0.831	** < 0.001**	0.657	0.940	81.8	76.2	64.3	88.9
NLR > 1.25	0.838	** < 0.001**	0.665	0.944	90.9	66.7	58.8	93.3
PLR > 7.78	0.905	** < 0.001**	0.748	0.980	90.9	81.0	71.4	94.4
SII > 339.46	0.879	** < 0.001**	0.715	0.967	100.0	61.9	57.9	100.0
PIV > 2524.23	0.745	**0.017**	0.560	0.881	72.7	76.2	61.5	84.2

Significant values are in bold.

## Discussion

In contrast to some studies suggesting that increased inflammatory response does not play a role in ADHD, the present study demonstrated that neutrophils, PLT, NLR, PLR, SII, and PIV levels were significantly higher in children with ADHD compared to the control group, while lymphocyte levels were significantly lower. ROC analysis revealed that these parameters had significant diagnostic values for ADHD, as indicated by the determined cut-off values. These findings are consistent with a study conducted by Abus et al., which reported higher SII and PIV levels in patients with obsessive–compulsive disorder compared to the control group^13^.

The findings of Akıncı & Uzun, which showed significantly higher neutrophil, PLT, and NLR levels in the ADHD group compared to the healthy group, are consistent with the results of the present study. However, unlike the present study, Akıncı & Uzun also observed elevated PDW, MPV, and WBC values in the ADHD group^1^. On the other hand, Aksu & Dağ reported a high MPV value in children with ADHD, particularly in boys, which contrasts the present study's findings 19]. Additionally, Avcil suggested in their study that the NLR ratio increased in ADHD and that the extent of NLR elevation played a role in the inflammatory pathophysiology of ADHD^9^.

The study conducted by Özdin et al. demonstrated higher NLR, PLR, and MLR values and lower lymphocyte numbers in patients with bipolar disorder and schizophrenia compared to the control group. Moreover, they observed increased platelet numbers in patients with bipolar disorder and elevated neutrophil numbers in patients with schizophrenia^8^. Similarly, Kirlioglu et al. reported higher PLR and MLR values in bipolar patients and elevated NLR and MLR values in manic patients^10^. These studies support the notion that inflammation markers, as reflected by NLR, PLR, and MLR, may play a role in the pathophysiology of these psychiatric conditions.

The study conducted by Ceyhun&Gürbüzer reported no significant difference in PDW between children in the ADHD group and the control group, which is consistent with the present study's findings. However, they mentioned that, unlike the present study, the SII ratio did not differ between the two groups^20^.

Similarly, the present study, in line with the study by Topal et al., found elevated neutrophil levels in children with ADHD, indicating consistency between the two studies. However, no significant difference in vitamin B12 and folate levels was observed in the present study, which differs from the findings of Topal et al.^14^.

Regarding examining CAR ratios in ADHD, the present study did not find a significant difference between children with ADHD and the control group of healthy children. It is essential to consider these variations in findings across studies and continue conducting further research to understand the role of inflammation markers and related factors in ADHD and other psychiatric conditions.

In the present study, no significant differences were found in MPV, PDW, and PCT levels between the ADHD group and the control group. However, Akbayram et al. reported high values for MPV, PDW, and PCT in children with ADHD, which contrasts with the present study's findings^15^.

Additionally, while Aksu & Dag reported gender-related differences in inflammatory marker levels in their study, no significant differences based on gender were observed for any parameter in the present study^19^.

Unlike C-reactive protein (CRP), albumin, an acute-phase reactant, decreases during inflammation^21^. While Lu et al. suggested that there might be low albumin levels in ADHD, this present study found no significant difference in albumin levels between children with ADHD and the healthy control group^16^.

However, a significant increase in albumin and lymphocyte levels was observed in the blood analysis conducted one month after treatment, compared to the pre-treatment blood analysis of children diagnosed with ADHD. This increase in albumin and lymphocyte levels could indicate a decrease in inflammation, the effectiveness of atomoxetine, or an improvement in ADHD symptoms. Consequently, albumin and lymphocyte levels can be considered parameters for ADHD prognosis. It is important to note that atomoxetine is highly bound to plasma albumin, with a binding rate of 99%^22^. Therefore, the observed increase in albumin and lymphocyte levels may be attributed to a direct mechanism of action independent of the drug's clinical effects.

The results of the present study are consistent with some previous studies. For example, Abus et al. reported higher SII and PIV levels in patients with obsessive–compulsive disorder, which aligns with the findings of the present study^13^. Similarly, Akıncı & Uzun found elevated neutrophil, PLT, and NLR levels in the ADHD group, supporting the results of the present study^1^. Avcil also suggested an increased NLR ratio in ADHD, indicating a potential role of inflammation in the pathophysiology of the disorder^9^.

However, there are also discrepancies between the present study and previous research. For instance, Aksu & Dağ reported elevated mean platelet volume (MPV), platelet distribution width (PDW), and white blood cell (WBC) values in the ADHD group, which were not observed in the present study. Aksu & Dağ also found gender-related differences in inflammatory marker levels, which were not significant in the present study^19^.

The inter-study differences in inflammation markers in ADHD may be attributed to various factors, including sample characteristics, study design, and measurement methods. The present study provides evidence of altered inflammatory markers in children with ADHD, supporting the involvement of inflammation in the disorder. The clinical significance of changes in these markers, such as the increase in albumin and lymphocyte levels after treatment, highlights their potential as prognostic indicators.

The fact that this study was conducted with a relatively small number of participants stands out as a major limitation. However, a purer patient group was obtained due to the exclusion of comorbid organic and mental illnesses of ADHD patients by the child psychiatrist and pediatrician. Evaluating inflammation markers only after one month will not be sufficient to evaluate the treatment of chronic diseases such as ADHD. Therefore, evaluating inflammation markers in the third and sixth months would be appropriate. The follow-up period may be extended in future studies. Additionally, it is a limitation that scales that evaluate disease severity, such as the Conners rating scale, are not used to evaluate response to treatment in ADHD patients.

## Conclusion

In this study, inflammation markers were higher in drug-naive ADHD patients than healthy controls. In addition, the improvement in lymphocyte and albumin values after atomoxetine treatment may be valuable in showing the decrease in the severity of inflammation. However, it would be more meaningful to evaluate ADHD patients after longer-term treatment and follow-up.

## Data Availability

The datasets used and analyzed during the current study are available from the corresponding author on reasonable request.

## Abbreviations

NLR: Neutrophil/lymphocyte ratio
PLR: Platelet/lymphocyte ratio
MLR: Monocyte/lymphocyte ratio
CAR: CRP/albumin ratio
SII: Systemic immune/inflammation index calculated as (platelet count × neutrophil count)/lymphocyte count
PIV: Pan-immune-inflammation value calculated as (platelet count × neutrophil count × monocyte count)/lymphocyte count
Hgb: Hemoglobin
WBC: White blood cell count
PDW: Platelet distribution width
PCT: Plateletcrit
